# Personals

**Published:** 1914-03

**Authors:** 


### PERSONALS

---

*Announcement of removal, travel, and other matters of interest are requested. Please report errors in the listing of any physician in the State and other directories that we may co-operate with the State Society in securing a correct list.*

---

Dr. A. L. Benedict would greatly appreciate information of sites of prehistoric Indian villages, discoveries of Indian graves, etc., in western New York.

---

Dr. Wm. H. Billings of Buffalo has recovered from an illness and is again attending to his practice.

---

Dr. Stephen Yates Howell of Buffalo, who returned from a trip around the world this winter, has located his office and residence at 501 Delaware avenue.

---

Dr. Edward H. Mehl has returned after having spent a year and a half in Europe. He was met in New York by his brother, Dr. Wm. H. Mehl.

---

Major Wm. H. Bissell, M. D., of Buffalo, recently retired after twenty-five years' surgical service in the 74th Regiment,

N. G. N. Y., has been honored by an unsolicited commission in the U. S. Medical Reserve Corps.

Dr. B. F. Senftenberg of New York has moved to 127 W. 82d street.

Dr. Joseph A. Gregory of Buffalo was married to Miss Susan Marie Elliott, February 17.

Drs. Roswell Park, Lucien Howe and Joseph Burke were recently appointed to the hospital committee of the Buffalo Chamber of Commerce.

Dr. Paul Sandreski of Buffalo had his automobile struck by an Erie switch engine at the Arthur street crossing of Tonawanda street, Buffalo, February 18. The automobile was wrecked. Dr. Sandreski was badly cut in the face and hands by glass from the wind shield.

